# Transmissible ST3-IncHI2 Plasmids Are Predominant Carriers of Diverse Complex IS*26*-Class 1 Integron Arrangements in Multidrug-Resistant *Salmonella*

**DOI:** 10.3389/fmicb.2018.02492

**Published:** 2018-10-23

**Authors:** Hang Zhao, Wenyao Chen, Xuebin Xu, Xiujuan Zhou, Chunlei Shi

**Affiliations:** ^1^State Key Laboratory of Microbial Metabolism, MOST-USDA Joint Research Center for Food Safety, School of Agriculture and Biology, Shanghai Jiao Tong University, Shanghai, China; ^2^Shanghai Municipal Center for Disease Control & Prevention, Shanghai, China

**Keywords:** *Salmonella*, IS*26*, class 1 integron, multidrug resistance, IncHI2

## Abstract

Diverse mobile genetic elements (MGEs) including plasmids, insertion sequences, and integrons play an important role in the occurrence and spread of multidrug resistance (MDR) in bacteria. It was found in previous studies that IS*26* and class 1 integrons integrated on plasmids to speed the dissemination of antibiotic-resistance genes in *Salmonella*. It is aimed to figure out the patterns of specific genetic arrangements between IS*26* and class 1 integrons located in plasmids in MDR *Salmonella* in this study. A total of 74 plasmid-harboring *Salmonella* isolates were screened for the presence of IS*26* by PCR amplification, and 39 were IS*26*-positive. Among them, 37 isolates were resistant to at least one antibiotic. The thirty-seven antibiotic-resistant isolates were further involved in PCR detection of class 1 integrons and variable regions, and all were positive for class 1 integrons. Six IS*26*-class 1 integron arrangements with IS*26* inserted into the upstream or downstream of class 1 integrons were characterized. Eight combinations of these IS*26*-class 1 integron arrangements were identified among 31 antibiotic-resistant isolates. Multidrug-resistance plasmids of the IncHI2 incompatibility group were dominant, which all belonged to ST3 by plasmid double locus sequence typing. These 21 IncHI2-positive isolates harbored six complex IS*26*-class 1 integron arrangement patterns. Conjugation assays and Southern blot hybridizations confirmed that conjugative multidrug-resistance IncHI2 plasmids harbored the different complex IS*26*-class 1 integron arrangements. The conjugation frequency of IncHI2 plasmids transferring alone was 10^−5^-10^−6^, reflecting that different complex IS*26*-class 1 integron arrangement patterns didn't significantly affect conjugation frequency (*P* > 0.05). These data suggested that class 1 integrons represent the hot spot for IS*26* insertion, forming diverse MDR loci. And ST3-IncHI2 was the major plasmid lineage contributing to the horizontal transfer of composite IS*26*-class 1 integron MDR elements in *Salmonella*.

## Introduction

*Salmonella* is recognized worldwide as a predominant pathogen causing foodborne diseases in humans (Yang et al., 2016). Multidrug resistance (MDR) among *Salmonella* toward numerous first-line agents, especially fluoroquinolones and extended-spectrum cephalosporins (ESCs) that are recommended as primary treatment choices for severe infections, may jeopardize therapy options and reduce the effectiveness of invasive Salmonellosis treatment (Folster et al., 2015; Tadesse et al., 2016). The recruitment, dissemination and rapid evolution of diverse antibiotic resistance in bacteria has been largely manipulated by mobile genetic elements (MGEs) such as plasmids, insertion sequences (ISs), transposons (Tns) and integrons via horizontal gene transfer (HGT) (Brown-Jaque et al., 2015). A typical example is that *Acinetobacter baumannii* isolates with a plasmid bearing IS*Aba1*-*bla*_*OXA*−51__−like_ gene had higher rates of resistance to imipenem and meropenem than those with the genes chromosomally encoded, probably due to increased gene dosage via higher copy number of associated plasmids (Chen et al., 2010).

Integrons are DNA elements capable of capturing and mobilizing exogenously functional gene cassettes, potentially permitting rapid adaptation to selective pressure and endowing increased fitness to the host (Deng et al., 2015). The class 1 integron is the most prevalent type associated with MDR *Salmonella*, playing a critical role in the dissemination of antibiotic resistance among various bacterial species (Li R. et al., 2013; Abraham et al., 2014). In addition, other MGEs could serve as vast reservoirs and massive genetic pool for integrons, facilitating their further extensive distribution (Sunde et al., 2015). ISs are the simplest autonomous mobile elements capable of transposing and altering the expression of neighboring genes (Siguier et al., 2014). IS*26* in multiple copies frequently reside in MDR plasmids flanking antibiotic resistance genes, performing actively in the fusion and reorganization of different plasmid replicons as well as the creation and diffusion of various MDR regions via a replicative mechanism or a translocatable unit (TU) (Harmer et al., 2014; He et al., 2015; García et al., 2016). It's noteworthy that IS*26* has been discovered to insert into and rearrange class 1 integrons, generating novel multi-resistance loci embedded in conjugative plasmids (Miriagou et al., 2005; Povilonis et al., 2010; Lai et al., 2013). The transposition activity of IS*26* collaborates with capture and integration of class 1 integrons, resembling resistance gene clusters onto a single plasmid and resulting in the occurrence and spread of MDR. Unfortunately, there is very little research directly targeting on the correlation between IS*26* and the class 1 integron in *Salmonella*, providing little highlights to trace IS*26*-class 1 integron-mediated MDR transmission and the evolution of MDR *Salmonella* under antibiotic selective pressure.

In this study, we analyzed IS*26* prevalence, antimicrobial resistance, class 1 integrons, and complex IS*26*-class 1 integron arrangements as well as their transfer functionality among *Salmonella* isolates. The objective of this study was to figure out regularity of specific genetic arrangements between IS*26* and class 1 integrons in *Salmonella* as well as to clarify the molecular mechanism of transferable IS*26*-class 1 integron-mediated MDR.

## Materials and methods

### *Salmonella* isolates

A total of 74 plasmid-harboring *Salmonella* isolates were used in this study, of which 37 were food isolates and 37 were clinical isolates. Among these *Salmonella* isolates, clinical isolates were collected by Shanghai Municipal Center for Disease Control and Prevention and Wuhan Municipal Center for Disease Control and Prevention, while food isolates were collected from beef, poultry, pork, shrimp, vegetables, fresh juice, and shellfish. Identification of plasmids by PCR-based replicon typing (PBRT) has been investigated by Chen et al. (2016). The detailed information of these isolates is listed in Table S1.

### Screening of IS*26*-positive isolates

Genomic DNA of *Salmonella* isolates was extracted by the TIANamp Genomic DNA kit (Tiangen Biotech, Beijing, China). All 74 *Salmonella* isolates were screened for the presence of IS*26* by simplex PCR amplification, according to Rodríguezmartínez et al. (2013).

### Antimicrobial susceptibility testing

The resulting IS*26*-positive *Salmonella* isolates underwent antimicrobial susceptibility testing using the disk diffusion method against a panel of 21 antibiotics, according to the standards and guidelines recommended by the Clinical and Laboratory Standards Institute (CLSI) (CLSI, 2013). A total of 21 antibiotic disks (Oxoid Ltd., Basingstoke, UK) that included ampicillin (AMP, 10 μg), piperacillin/tazobactam (TZP, 100/10 μg), ampicillin/sulbactam (SAM, 10/10 μg), ceftriaxone (CRO, 30 μg), ceftazidime (CAZ, 30 μg), cefepime (FEP, 30 μg), cefotetan (CTT, 30 μg), aztreonam (ATM, 30 μg), cephazolin (CZO, 30 μg), ciprofloxacin (CIP, 5 μg), imipenem (IPM, 10 μg), amikacin (AMK, 30 μg), gentamicin (GEN, 10 μg), tobramycin (TOB, 10 μg), ertapenem (ETP, 10 μg), levofloxacin (LEV, 5 μg), nitrofurantoin (NIT, 300 μg), sulfamethoxazole/trimethoprim (SXT, 23.75/1.25 μg), streptomycin (STR, 10 μg), chloramphenicol (CHL, 30 μg), and tetracycline (TET, 30 μg) were assessed. *Escherichia coli* ATCC 25922 was used as control strain. Isolates were defined as MDR if they were resistant to at least three different classes of antibiotics.

### Detection of class 1 integrons

The presence of class 1 integrons was determined by conventional PCR targeting the class 1 integrase gene *intI1* and the *qacE*Δ*1-sulI* genes in the 3′-conserved segment (3′CS) using primers *intI1*-F/*intI1*-R and QS-F/QS-R respectively listed in Table S2 among IS*26*-positive antibiotic-resistant *Salmonella* isolates. Primers 5′CS/*qacE*Δ*1*R, 5′CS/3′CS, hep58/hep59, and 5′CS/hep59 (see Table S2) were then used to amplify gene cassettes within the variable region of class 1 integrons by a touch-down PCR protocol (annealing temperature decreasing from 60 to 50°C in 20 cycles, and then 15 cycles at 50°C). PCR products were purified using the AxyPrep DNA Gel Extraction Kit (Axygen, USA) and sequenced by Shanghai Majorbio Bio-pharm Technology Co., Ltd. Comparative analysis of nucleotide sequences was performed using the BLAST program at the National Center for Biotechnology Information (NCBI) site (http://blast.ncbi.nlm.nih.gov/Blast).

### Genetic context analysis of class 1 integrons associated with IS*26*

**Figure 1:**
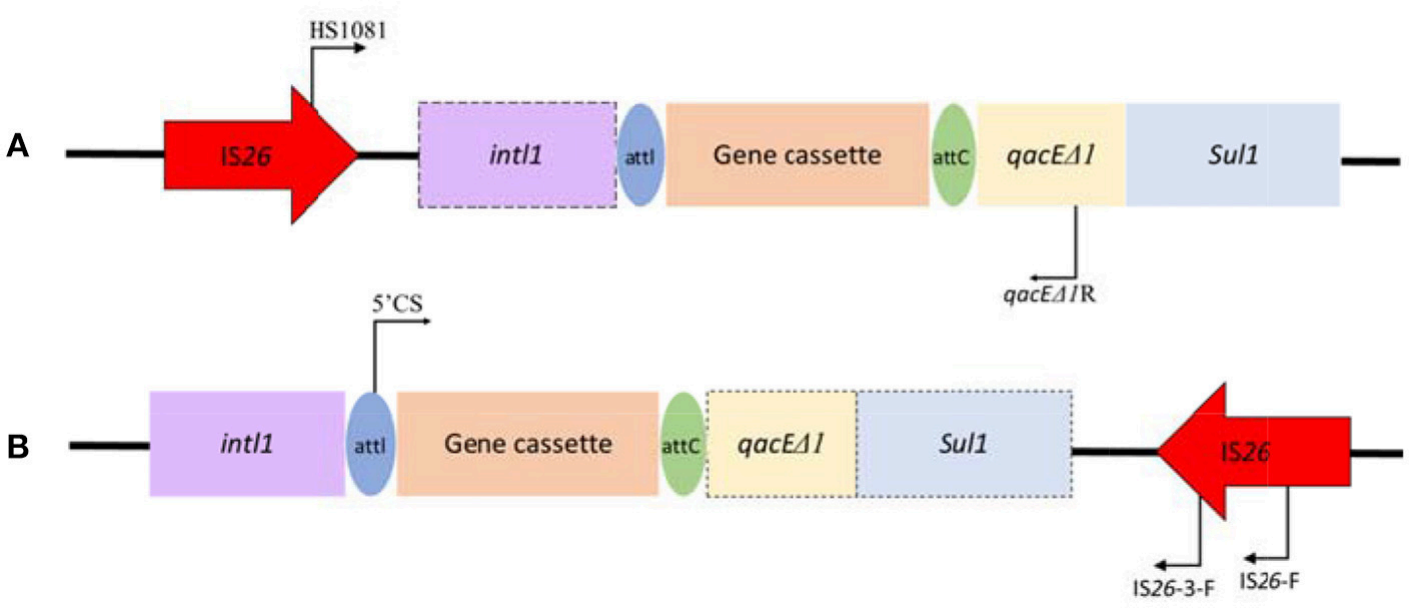
Schematic representation of genetic association between IS*26* and class 1 integrons toward their frequently reported position relationship with IS*26* inserted into **(A)** the upstream or **(B)** the downstream of class 1 integrons. The dashed frames indicate the genes of the class 1 integron that may be truncated by IS*26*. The locations of the primers used for the PCR assay are represented with dark arrows.

Genetic context associated with IS*26* and class 1 integrons toward their frequently reported position relationship, was carried out by a touch-down PCR protocol (annealing temperature decreasing from 65 to 55°C in 20 cycles, and then 15 cycles at 50°C) among *Salmonella* isolates both positive for IS*26* and class 1 integrons. Primers used were also listed in Table S2. Primers HS1081/*qacE*Δ*1R* targeted the IS*26*-class 1 integron relationship with the IS inserted into the upstream of class 1 integron, while primers 5′CS/IS*26*-F and 5′CS/IS*26*-3-F targeted the position relationship with the IS*26* inserted into the downstream of class 1 integrons (Figure 1). PCR products were purified using the AxyPrep DNA Gel Extraction Kit (Axygen, USA) and sequenced by Shanghai Majorbio Bio-pharm Technology Co., Ltd. Comparative analysis of nucleotide sequences was performed using the BLAST program at the NCBI site (http://blast.ncbi.nlm.nih.gov/Blast).

### IncHI2 plasmid characterization and conjugation experiment

IncHI2 was dominant incompatibility group in this study (Table S1). To better characterize IncHI2 plasmids, plasmid double locus sequence typing (pDLST) was performed as previously described (García-Fernández and Carattoli, 2010). To investigate the association between IncHI2 plasmids and complex IS*26*-class 1 integron arrangements, the corresponding isolates harboring both elements underwent the liquid mating assay (Dang et al., 2016) using rifampin-resistant *E. coli* NK5449 as the recipient strain. Transconjugants were selected on LB agar plates supplemented with rifampin (200 μg/ml) and another appropriate antibiotic [kanamycin (50 μg/ml), streptomycin (50 μg/ml), tetracycline (50 μg/ml), or ciprofloxacin (16 μg/ml)]. Conjugation frequencies were also calculated as the number of transconjugants per recipient for several representative isolates. The putative transconjugants were examined for the antibiotic susceptibility profile using the same set of antibiotics, and for the presence of complex IS*26*-class 1 integron arrangements as well as plasmid replicon type by PCR method as described above.

### Confirmation of the IS*26*-class 1 integron arrangements located on IncHI2 plasmids

The transconjugants harboring both IncHI2 plasmids and typical complex IS*26*-class 1 integron arrangement patterns were selected to determine the plasmid size and location of those typical complex arrangements. All types of IS*26*-class 1 integron arrangements and IncHI2 replicon were PCR amplified (Table 1) and then purified using Axyprep DNA gel extraction kit (Axygen, Corning, China). All PCR amplifications were performed with the following amplification scheme: 1 cycle of denaturation at 94°C for 5 min, followed by 35 cycles of denaturation at 94°C for 1 min, annealing at different annealing temperature for 30 s and elongation at 72°C for 1 min. The amplification was concluded with an extension program of 1 cycle at 72°C for 10 min. The purified PCR products were labeled by DIG High Prime DNA Labeling and Detection Starter Kit I (Roche Applied Sciences, Germany) to be later used as Southern blot probes. The total DNA of transconjugants was first prepared in agarose plugs, digested with S1 nuclease (TaKaRa, China) and further separated by PFGE using CHEF-Mapper XA PFGE system (Bio-Rad, USA) to distinguish the plasmids of transconjugants (Dierikx et al., 2010). *S*. Braenderup H9812 universal size standard was used as PFGE marker (Hunter et al., 2005). The separated DNA fragments were transferred to a nylon membrane (Amersham, GE, USA), and then hybridized with the IncHI2 probe and corresponding digoxigenin-labeled IS*26*-class 1 integron arrangement probes, and finally detected using a NBT/BCIP color detection kit according to the manufacturer's instructions (Roche Applied Sciences, Germany).

**Table 1:** Specific digoxigenin-labeled IncHI2 and IS*26*-class 1 integron arrangement probes.

**No**.	**Digoxigenin-labeled probe**	**Primer sequence (5′ → 3′)**	**PCR reaction annealing temperature (°C)**	**Probe Size (bp)**
I	IS*26-bla*_OXA−1_ *-catB3-arr3-*3′CS	F: AGCCCTTTACCAAACCAA	56	395
		R: CGAAACCCAAACAACAGA		
II	IS*26-aac(6′)-Ib-cr-bla*_OXA−1_*-catB3-arr3-*3′CS	F: TTGCGATGCTCTATGAGTGGCTA	58	482
		R: CTCGAATGCCTGGCGTGTTT		
III	5′CS-*dfrA12*-*orfF*-Δ*aadA2*-IS*26*	F: ACTGGCTGCGTAGTTGTT	52	183
		R: GTTGAGCATTGGGAAGAA		
IV	5′CS*-estX-psp-aadA2-*Δ*cmlA1*(5′-524 bp truncated)-IS*26*	F: CGGGCTATCTTTGCGTTTC	55	101
		R: CGCCTGGTAAGCAGAGTTTT		
V	5′CS*-estX-psp-aadA2-*Δ*cmlA1*(3′-15 bp truncated)-IS*26*	F: TGATGGGCAGGCAAGGTG	57	384
		R: GCGGCAACAGCGAAATGA		
HI2	IncHI2 iteron	F: TTTCTCCTGAGTCACCTGTTAACAC	60	644
		R: GGCTCACTACCGTTGTCATCCT		

### Nucleotide sequence accession number

The nucleotide sequences of gene cassette arrays embedded in class 1 integrons found in this study have been deposited in GenBank/EMBL/DDBJ under the following accession numbers: KY399735 (*dfrA12-orfF-*Δ*aadA2-*IS*26-*Δ*Tn3-orf*), KY399738 (*dfrA17-aadA5*-IS*26*), KY399736 (*dfrA12-orfF-aadA2*), and KY399737 (*dfrA17-aadA5*). The nucleotide sequences of characterized complex IS*26*-class 1 integron arrangements have also been deposited in GenBank/EMBL/DDBJ under the following accession numbers: KY399739 (IS*26-aac(6*′*)-Ib-cr-bla*_OXA−1_*-catB3-arr3-*3′CS), KY399740 (IS*26-bla*_OXA−1_ *-catB3-arr3-*3′CS), KY399741 (IS*26-*Δ*tnpR-tnpM-intI1-dfrA17-aadA5-*3′CS), KY399744 (5′CS*-estX-psp-aadA2-*Δ*cmlA1*(5′-524 bp truncated)-IS*26*), KY399743 (5′CS*-estX-psp-aadA2-*Δ*cmlA1*(3′-15 bp truncated)-IS*26*), and KY399742 (5′CS-*dfrA12-orfF-*Δ*aadA2*-IS*26*).

## Results and discussion

### IS*26* prevalence and antimicrobial susceptibility

**Figure 2:**
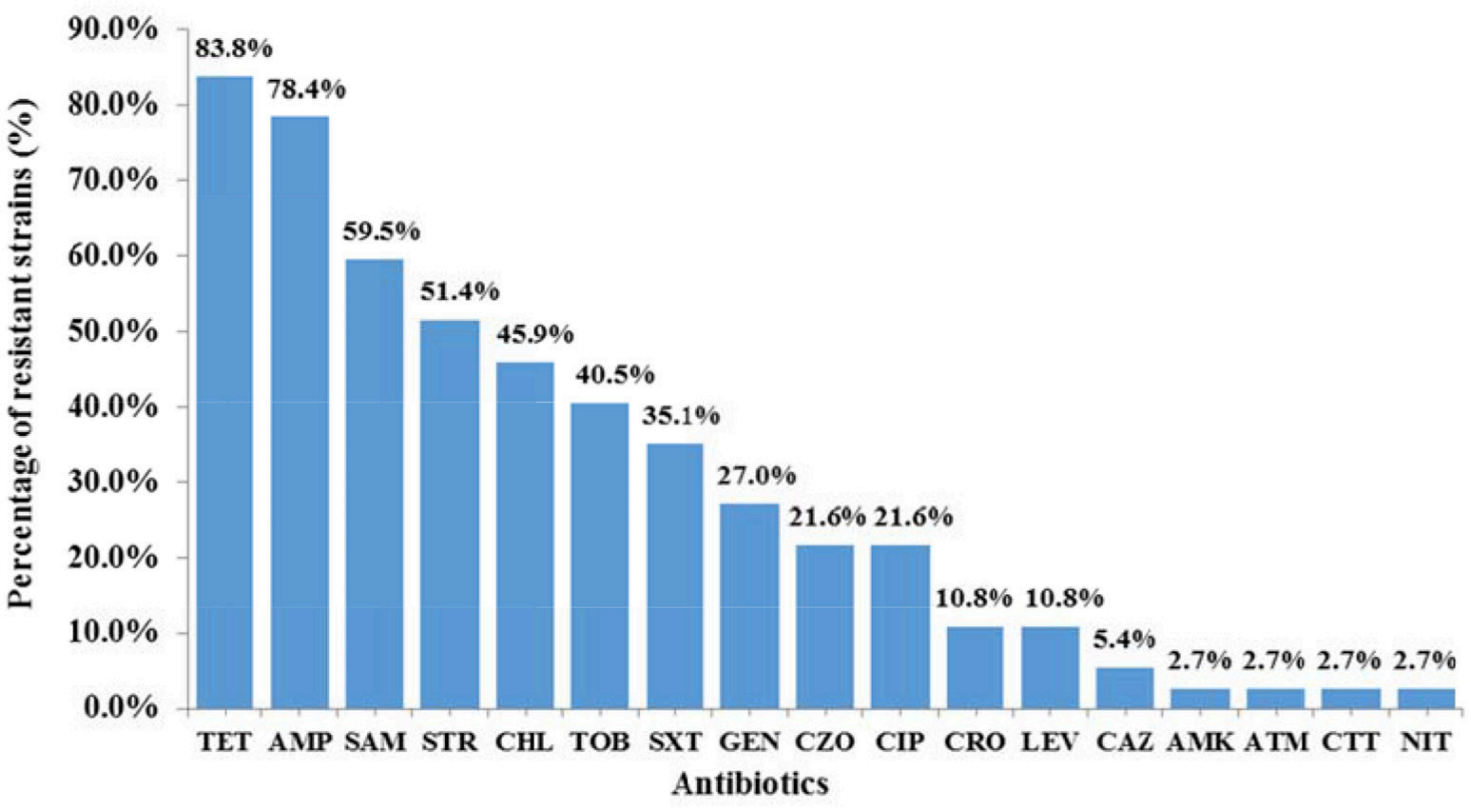
The resistance to individual agents among 37 IS*26*-carrying antibiotic-resistant *Salmonella* isolates. The antibiotics listed are abbreviated as follows: AMK, amikacin; AMP, Ampicillin; ATM, aztreonam; CAZ, ceftazidime; CHL, chloramphenicol; CIP, ciprofloxacin; CRO, ceftriaxone; CTT, cefotetan; CZO, cephazolin; GEN, gentamicin; LEV, levofloxacin; NIT, nitrofurantoin; SAM, ampicillin/sulbactam; STR, streptomycin; SXT, sulfamethoxazole/trimethoprim; TET, tetracycline; TOB, tobramycin.

IS*26* was present in 52.7% (39/74) of plasmid-harboring *Salmonella* isolates. Amongst the 39 IS*26*-positive isolates, 94.9% (37/39) demonstrated resistance to at least one antibiotic, of which 70.3% (26/37) showed MDR phenotypes. It's noteworthy that the strain SJTUF 10702 isolated from chicken exhibited resistance to 14 antibiotics. Among the 37 antibiotic-resistant isolates (Figure 2), resistance to individual agents was most frequently observed against TET (83.8%) and AMP (78.4%), followed by SAM (59.5%) and STR (51.4%). Resistance to CHL (45.9%), TOB (40.5%), SXT (35.1%), GEN (27.0%), CZO (21.6%), CIP (21.6%), CRO (10.8%), LEV (10.8%), CAZ (5.4%), AMK (2.7%), ATM (2.7%), CTT (2.7%), and NIT (2.7%) were less common. No resistance was detected to TZP, FEP, IPM, and ETP.

### Characterization of class 1 integrons

**Figure 3:**
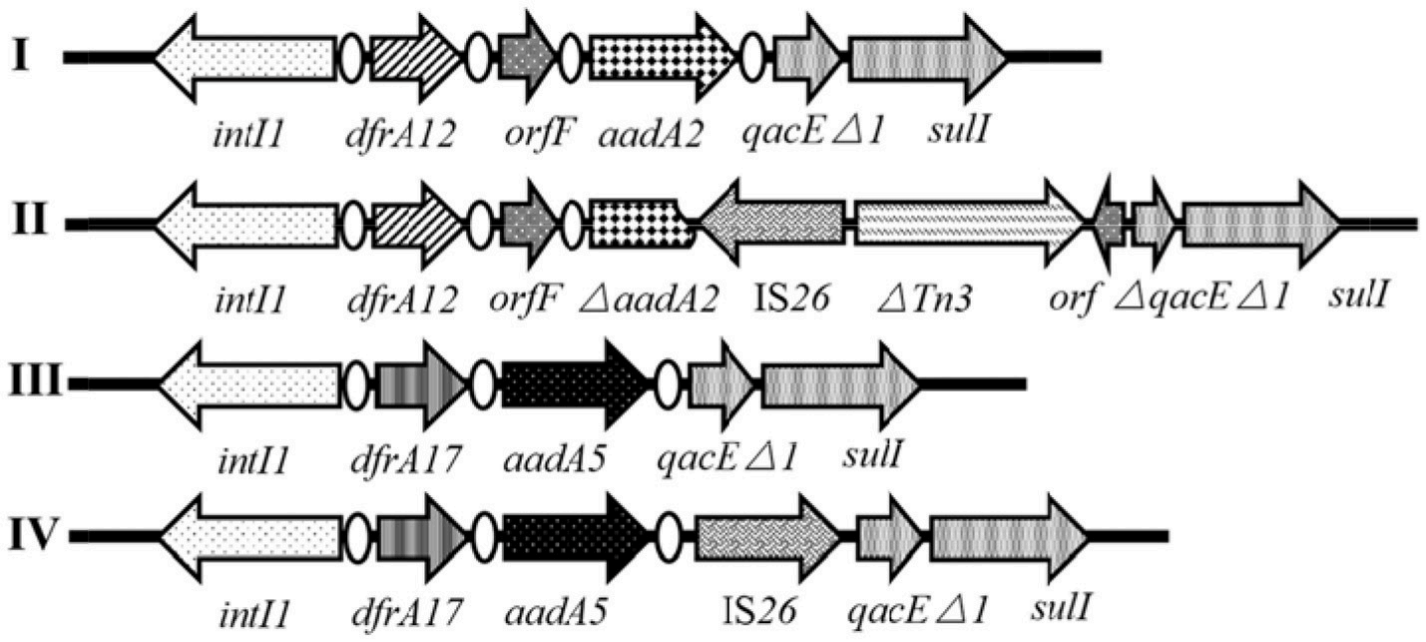
Genetic organization of different class 1 integrons. The orientation of each gene and insertion element is indicated by arrows.

Both *intI1* and *qacE*Δ*1-sulI* genes of the class 1 integron were detected in all of the 37 IS*26*-positive antibiotic-resistant *Salmonella* isolates, of which 16 (43.2%) isolates harbored variable regions clustered in four different cassette arrays (Figure 3, I~IV). Apart from *qacE*Δ*1* and *sulI* genes responsible for resistance to quaternary ammonium compounds and sulfonamides, respectively, the four antibiotic resistance gene cassettes confer resistance to aminoglycosides with *aadA2* or *aadA5*, and confer resistance to trimethoprim with *dfrA12* or *dfrA17*. Two such cassette arrays were embedded in simple integrons consisting of *dfrA12*-*orfF-aadA2* (1.9 kb, *n* = 4, Figure 3I) and *dfrA17-aadA5* (1.6 kb, *n* = 4, Figure 3III), while the other two were embedded in complex integrons carrying the IS*26* element consisting of *dfrA17-aadA5*-IS*26* (2.5 kb, *n* = 1, Figure 3IV) and *dfrA12-orfF-*Δ*aadA2*-IS*26*-Δ*Tn3-orf* (4 kb, *n* = 7, Figure 3II). Array I and III in Figure 3 were popularly distributed in *Salmonella* (Li R. et al., 2013; Pérez-Moreno et al., 2013; Meng et al., 2017). Compared to Array I, *aadA2* gene in Array II was truncated at the 578-bp from the 5′ CS by IS*26*, along with the insertion of a Δ*Tn3-orf* fragment and partial deletion of the *qacE*Δ*1* gene. Compared to Array III, reversely oriented IS*26* inserted into the downstream of the *aadA5* gene in Array IV without any disruption.

### Characterization of complex IS*26*-class 1 integron arrangements

Toward the position relationship with IS*26* inserted into the upstream of class 1 integrons, three different IS*26*-class 1 integron arrangements were characterized as follows: IS*26-aac(6*′*)-Ib-cr-bla*_OXA−1_*-catB3-arr3-*3′CS (3.5 kb, *n* = 22), IS*26-bla*_OXA−1_ *-catB3-arr3-*3′CS (2.8 kb, *n* = 2), and IS*26-*Δ*tnpR-tnpM-intI1-dfrA17-aadA5-*3′CS (4 kb, *n* = 2) [Table 2, IS26(→)-3′CS]. Toward the position relationship with IS*26* inserted into the downstream of class 1 integrons, three different IS*26*-class 1 integron arrangements were also characterized as follows: 5′CS-*dfrA12-orfF-*Δ*aadA2*-IS*26* (2.3 kb, *n* = 24), 5′CS*-estX-psp-aadA2-*Δ*cmlA1*(3′-15 bp truncated)-IS*26* (4.3 kb, *n* = 1), and 5′CS*-estX-psp-aadA2-*Δ*cmlA1*(5′-524 bp truncated)-IS*26* (3.5 kb, *n* = 4) [Table 2, 5′CS-IS26(←)]. Among the 37 antibiotic-resistant isolates, only 31 were positive for complex IS*26*-class 1 integron arrangements mentioned above with eight different complex patterns shown in Table 2.

**Table 2:** Complex IS*26*-class 1 integron arrangement patterns characterized in 31 antibiotic-resistant *Salmonella* isolates, including isolated year, sources, serovar, antibiotic resistance profiles, complex IS*26*-class 1 integron arrangements as well as plasmid replicon types.

**Isolate**	**Year**	**Origin**	**Serovar**	**IS*26*(→)-3′CS**	**5′CS-IS*26*(←)**	**Replicon**	**Resistance pattern**
SJTUF 10231	2007	Feces	Typhimurium	IS*26-aac(6′)-Ib-cr-bla*_OXA−1_*-catB3-arr3-*3′CS	5′CS-*dfrA12*-*orfF*-*ΔaadA2*-IS*26*	HI2	AMP, SAM, STR, TOB, TET
SJTUF 10157	2006	Feces	Typhimurium	IS*26-aac(6′)-Ib-cr-bla*_OXA−1_*-catB3-arr3-*3′CS	5′CS-*dfrA12*-*orfF*-*ΔaadA2*-IS*26*	HI2	AMP, SAM, STR, SXT, CHL, TET
SJTUF 10236	2007	Feces	Typhimurium	IS*26-aac(6′)-Ib-cr-bla*_OXA−1_*-catB3-arr3-*3′CS	5′CS-*dfrA12*-*orfF*-*ΔaadA2*-IS*26*	HI2	AMP, SAM, TOB, SXT, CHL, TET
SJTUF 10580	2006	Pork	Anatum	IS*26-aac(6′)-Ib-cr-bla*_OXA−1_*-catB3-arr3-*3′CS	5′CS-*dfrA12*-*orfF*-*ΔaadA2*-IS*26*	HI2	AMP, SAM, GEN, TOB, SXT, CHL
SJTUF 10057	2006	Feces	Typhimurium	IS*26-aac(6′)-Ib-cr-bla*_OXA−1_*-catB3-arr3-*3′CS	5′CS-*dfrA12*-*orfF*-*ΔaadA2*-IS*26*	HI2	AMP, SAM, GEN, STR, TOB, SXT, CHL, TET
SJTUF 10578	2006	Pork	Typhimurium	IS*26-aac(6′)-Ib-cr-bla*_OXA−1_*-catB3-arr3-*3′CS	5′CS-*dfrA12*-*orfF*-*ΔaadA2*-IS*26*	HI2	AMP, SAM, GEN, STR, TOB, SXT, CHL, TET
SJTUF 10476	2007	Chicken	Indiana	IS*26-aac(6′)-Ib-cr-bla*_OXA−1_*-catB3-arr3-*3′CS	5′CS-*dfrA12*-*orfF*-*ΔaadA2*-IS*26*	HI2	AMP, SAM, GEN, STR, TOB, CIP, LEV, SXT, CHL, TET
SJTUF 10169	2006	Feces	Typhimurium	IS*26-aac(6′)-Ib-cr-bla*_OXA−1_*-catB3-arr3-*3′CS	5′CS-*dfrA12*-*orfF*-*ΔaadA2*-IS*26*	HI2, N	AMP, TOB, SXT, CHL, TET
SJTUF 10112	2006	Feces	Typhimurium	IS*26-aac(6′)-Ib-cr-bla*_OXA−1_*-catB3-arr3-*3′CS	5′CS-*dfrA12*-*orfF*-*ΔaadA2*-IS*26*	HI2, N	AMP, STR, TOB, SXT, CHL, TET
SJTUF 10584	2006	Chicken	Indiana	IS*26-aac(6′)-Ib-cr-bla*_OXA−1_*-catB3-arr3-*3′CS	5′CS-*dfrA12*-*orfF*-*ΔaadA2*-IS*26*	HI2, I1	AMP, SAM, CAZ, CRO, CZO, CIP, SXT, CHL, TET
SJTUF 10484	2007	Clam	Typhimurium	IS*26-aac(6′)-Ib-cr-bla*_OXA−1_*-catB3-arr3-*3′CS	5′CS-*dfrA12*-*orfF*-*ΔaadA2*-IS*26*	HI2, A/C, P	AMP, TET
SJTUF 10229	2007	Feces	Enteritidis	IS*26-aac(6′)-Ib-cr-bla*_OXA−1_*-catB3-arr3-*3′CS	5′CS-*dfrA12*-*orfF*-*ΔaadA2*-IS*26*	FIIS	AMP, SAM, STR, TET, NIT
SJTUF 10573	2006	Razor clam	Stanley	IS*26-aac(6′)-Ib-cr-bla*_OXA−1_*-catB3-arr3-*3′CS	5′CS-*dfrA12*-*orfF*-*ΔaadA2*-IS*26*	P	SXT, TET
SJTUF 10469	2007	Pork	Derby	IS*26-aac(6′)-Ib-cr-bla*_OXA−1_*-catB3-arr3-*3′CS	5′CS-*dfrA12*-*orfF*-*ΔaadA2*-IS*26*	P, FIC	TET
SJTUF 10211	2007	Feces	Typhimurium	ND	5′CS-*dfrA12*-*orfF*-*ΔaadA2*-IS*26*	HI2, P	AMP, SAM, STR, SXT, TET
SJTUF 10585	2006	Chicken	Indiana	ND	5′CS-*dfrA12*-*orfF*-*ΔaadA2*-IS*26*	HI2, I1	AMP, SAM, CAZ, CRO, CZO, CIP, LEV, TET
SJTUF 10456	2007	Pork	Derby	ND	5′CS-*dfrA12*-*orfF*-*ΔaadA2*-IS*26*	HI2, P, N, FIC	TET
SJTUF 10718	2006	Feces	Enteritidis	ND	5′CS-*dfrA12*-*orfF*-*ΔaadA2*-IS*26*	FIIS	AMP, GEN, STR, TOB, TET
SJTUF 10207	2007	Feces	Indiana	ND	5′CS-*dfrA12*-*orfF*-*ΔaadA2*-IS*26*	P, N	CIP
SJTUF 10475	2007	Pork	Derby	ND	5′CS-*dfrA12*-*orfF*-*ΔaadA2*-IS*26*	I1, P, FIC	TET
SJTUF 10570	2006	Pork	Typhimurium	IS*26-aac(6′)-Ib-cr*-*bla*_OXA−1_-*catB3*-*arr3-*3′CS	ND	HI2	AMP, SAM, STR, TET
SJTUF 10702	2006	Chicken	Indiana	IS*26-aac(6′)-Ib-cr*-*bla*_OXA−1_-*catB3*-*arr3-*3′CS	ND	P	AMP, SAM, ATM, CRO, CZO, AMK, GEN, STR, TOB, CIP, LEV, SXT, CHL, TET
SJTUF 10250	2007	Feces	Typhimurium	IS*26-aac(6′)-Ib-cr*-*bla*_OXA−1_-*catB3*-*arr3-*3′CS	5′CS-*estX-psp-aadA2-ΔcmlA1(*5′-524 bp truncated)-IS*26*	HI2	AMP, SAM, STR, TOB, CHL, TET
SJTUF 10330	2007	Feces	Typhimurium	IS*26-aac(6′)-Ib-cr*-*bla*_OXA−1_-*catB3*-*arr3-*3′CS	5′CS-*estX-psp-aadA2-ΔcmlA1*(5′-524 bp truncated)-IS*26*	HI2	AMP, SAM, GEN, STR, TOB, CHL, TET
SJTUF 10568	2006	Pork	Typhimurium	IS*26-aac(6′)-Ib-cr*-*bla*_OXA−1_-*catB3*-*arr3-*3′CS	5′CS-*estX-psp-aadA2-ΔcmlA1*(5′-524 bp truncated)-IS*26*	HI2	AMP, SAM, GEN, STR, TOB, CHL, TET
SJTUF 10567	2006	Pork	Typhimurium	IS*26-aac(6′)-Ib-cr*-*bla*_OXA−1_-*catB3*-*arr3-*3′CS	5′CS-*estX-psp-aadA2-ΔcmlA1*(5′-524 bp truncated)-IS*26*	HI2, A/C	AMP, SAM, STR, TOB, CHL, TET
SJTUF 10565	2006	Chicken	Typhimurium	IS*26-aac(6′)-Ib-cr*-*bla*_OXA−1_-*catB3*-*arr3-*3′CS	5′CS*-estX-psp-aadA2-ΔcmlA1(*3′-15 bp truncated)-IS*26*	HI2	AMP, SAM, CZO, GEN, TOB, CHL
SJTUF 10772	2006	Feces	Heidelberg	IS*26-ΔtnpR-tnpM-intI1-dfrA17-aadA5-*3′CS	5′CS-*dfrA12*-*orfF*-*ΔaadA2*-IS*26*	P	AMP, CZO
SJTUF 10713	2006	Chicken	Heidelberg	IS*26-ΔtnpR-tnpM-intI1-dfrA17-aadA5-*3′CS	5′CS-*dfrA12*-*orfF*-*ΔaadA2*-IS*26*	FIA, FIB	AMP, SAM, CZO, GEN, CIP, LEV, CHL, TET
SJTUF 10703	2007	Shrimp	Thompson	IS*26-bla*_OXA−1_*-catB3-arr3-*3′CS	5′CS-*dfrA12*-*orfF*-*ΔaadA2*-IS*26*	A/C	AMP, SAM, CRO, CZO, STR, CIP, SXT, CHL, TET
				IS*26-aac(6′)-Ib-cr-bla_*OXA*−1_-catB3-arr3-*3′CS			
SJTUF 10577	2006	Saury	Typhimurium	IS*26-bla*_OXA−1_*-catB3-arr3-*3′CS	5′CS-*dfrA12*-*orfF*-*ΔaadA2*-IS*26*	HI2, P	AMP, STR, TET

*AMP, ampicillin; SAM, ampicillin/sulbactam; CAZ, ceftazidime; CRO, ceftriaxone; CZO, cephazolin; GEN, gentamicin; STR, streptomycin; TOB, tobramycin; CIP, ciprofloxacin; LEV, levofloxacin; SXT, sulfamethoxazole/trimethoprim; CHL, chloramphenicol; TET, tetracycline; ATM, aztreonam; AMK, amikacin. ND, non-detected*.

*Yellow, IS26-blaOXA-1-catB3-arr3-3′CS; green, IS26-aac(6′)-Ib-cr-blaOXA-1-catB3-arr3-3′CS; red, 5′CS-dfrA12-orfF-ΔaadA2-IS26; blue, IS26-ΔtnpR-tnpM-intI1-dfrA17-aadA5-3′CS; purple, 5′CS-estX-psp-aadA2-ΔcmlA1(5′-524 bp truncated)-IS26; gray, 5′CS-estX-psp-aadA2-ΔcmlA1(3′-15 bp truncated)-IS26*.

The genetic arrangement of IS*26-aac(6*′*)-Ib-cr-bla*_OXA−1_*-catB3-arr3-*3′CS was composite structure consisting of an IS*26* element and a peculiar class 1 integron without 5′CS, which carrying *aac(6*′*)-Ib-cr, bla*_OXA−1_, *catB3, arr3* gene cassettes, so conferring resistance to quinolone and aminoglycoside, ampicillin, chloramphenicol, and rifampicin, respectively. The Pc promoter responsible for the expression of gene cassettes is located in the 5′CS region of the class 1 integron (Stalder et al., 2012). Interestingly, 17 of 22 isolates carrying this genetic arrangement exhibited antibiotic tolerance against ampicillin and chloramphenicol (Table 2, green), suggesting that IS*26* achieved gene cassette expression through forming a suitable−10 box aided by−35 box in the IR of IS*26* (Lee et al., 1990; Cain and Hall, 2011). In comparison, another genetic arrangement of IS*26-bla*_OXA−1_*-catB3-arr3-*3′CS was similar but lacking the *aac(6*′*)-Ib-cr* gene cassette (Table 2, yellow). Interestingly, *S*. Thompson isolate SJTUF 10703 harboring both IS*26-aac(6*′*)-Ib-cr-bla*_OXA−1_*-catB3-arr3-*3′CS and IS*26-bla*_OXA−1_*-catB3-arr3-*3′CS conferred resistance to 9 antibiotics. *S*. Typhimurium isolate SJTUF 10577 simultaneously harboring IS*26-bla*_OXA−1_ *-catB3-arr3-*3′CS and 5′CS-*dfrA12-orfF-*Δ*aadA2*-IS*26* showed less antibiotic resistance than other *S*. Typhimurium isolates harboring IS*26-aac(6*′*)-Ib-cr-bla*_OXA−1_*-catB3-arr3-*3′CS and 5′CS-*dfrA12-orfF-*Δ*aadA2*-IS*26*, indicating that the emergence of IS*26-aac(6*′*)-Ib-cr-bla*_OXA−1_*-catB3-arr3-*3′CS may be the outcome of molecular evolution of IS*26-bla*_OXA−1_*-catB3-arr3-*3′CS under antibiotic pressure. Another novel composite structure was identified, consisting of an IS*26* element and a peculiar Tn*21* with *tnpR* gene (encoding resolvase) truncated by IS*26* while an intact class 1 integron was embedded in Tn*21* carrying the gene cassette array of *dfrA17-aadA5* (Table 2, blue). This arrangement was only found in two *S*. Heidelberg isolates from feces and chicken, respectively. Dawes et al. (2010) also discovered a complex IS*26*-Tn*21* module in *E. coli*. But in their findings, IS*26* directly inserted into the downstream of the *aadA5* gene cassette embedded in the class 1 integron, truncating the 3′CS and leaving functional genes of Tn*21* intact.

The IS*26*-class 1 integron arrangements with the IS*26* inserted into the downstream of class 1 integrons were all composite structures consisting of an IS*26* element and a peculiar class 1 integron with gene cassettes interrupted. On the basis of sequence alignments, the prevalent arrangement of 5′CS-*dfrA12-orfF-*Δ*aadA2*-IS*26* (Table 2, red) may originate from the typical class 1 integron with the array of *intI1*-*dfrA12-orfF-aadA2-qac*Δ*E-sulI* or the *sul3*-type class 1 integron with the array of *intI1*-*dfrA12-orfF-aadA2-cmlA1-aadA1-qacH-*IS*440-sul3* (Antunes et al., 2007), due to the insertion of IS*26* at the 578-bp of the *aadA2* gene cassettes from the 5′ end. Furthermore, The other two genetic arrangements of 5′CS*-estX-psp-aadA2-*Δ*cmlA1-*IS*26* (Table 2, purple and gray) may both originate from the *sul3*-type class 1 integron carrying the gene cassette array of *estX-psp-aadA2-cmlA1-aadA1* (Antunes et al., 2007), similarly due to the insertion of IS*26* at different loci of the *cmlA1* gene cassette (conferring resistance to chloramphenicol). Other studies also discovered the correlation between IS*26* and *sul3*-type class 1 integrons with IS*26* frequently inserted into the downstream of the *qacH-sul3* domain, forming IS*440*-*sul3*-Δ*orf1-*IS*26* clusters (Curiao et al., 2011; Moran et al., 2016). These data pointed out the potential of IS*26* to mediate horizontal transfer of *sul3*-type class 1 integrons.

Target site duplication (TSD) is the characteristic hallmark of transposition (He et al., 2015). However, 8-bp typical TSD (TTCTACGG) (Oliveira et al., 2013) of IS*26* transposition didn't occur among complex IS*26*-class 1 integron arrangements characterized in this study, which was also observed in some researches (Miriagou et al., 2005; Curiao et al., 2011; Hudson et al., 2014). Since IS*26* transposes via a cointegrating mechanism, homologous recombination following IS*26* transposition may cause DNA deletions or rearrangements, merely resulting in the generation of itself without flanking TSDs (He et al., 2015). In addition, IS*26* in the Translocatable Unit (TU) targets an existing copy of IS*26* and the TU will be incorporated immediately adjacent to it without increasing the number of IS*26* copies or creating a duplication of the target (Harmer et al., 2014). Thus, further complete sequencing and analysis of representative plasmids harboring specific complex IS*26*-class 1 integron arrangements may be required to better understand the molecular mechanism of IS*26*-class 1 integron-mediated MDR.

Eight complex IS*26*-class 1 integron arrangement patterns shown in Table 2 were distributed in 8 *Salmonella* serovars with the high prevalence of *S*. Typhimurium (51.6%, 16/31). And six complex IS*26*-class 1 integron arrangement patterns were distributed in the IncHI2-positive *Salmonella* isolates. *S*. Typhimurium is an important foodborne pathogen with a high prevalence of antimicrobial resistance (Torpdahl et al., 2013). All of *S*. Typhimurium isolates harboring complex IS*26*-class 1 integron arrangements except one exhibited MDR phenotypes, inferring that composite IS*26*-class 1 integron elements may play a critical role in the acquisition and dissemination of antibiotic resistance as well as the environmental adaptation of *S*. Typhimurium. Moreover, *S*. Typhimurium isolates with the same complex IS*26*-class 1 integron arrangement pattern showed similar antibiotic resistance profile, and vice versa (Table 2). The diversity of complex IS*26*-class 1 integron arrangement patterns may be useful as a marker in epidemiological studies of outbreak associated with *S*. Typhimurium that contain such elements, assisting in foodborne disease source-tracking and the antibiotic resistance surveillance.

### Characterization of IncHI2 plasmids and localization of the complex IS*26*-class 1 integron arrangement on IncHI2 plasmids

IncHI2 plasmids are responsible for carrying numerous classes of resistance genes and frequently detected among MDR *Salmonella* (Lai et al., 2013; Li L. et al., 2013; Li et al., 2014). IncHI2 was the dominant incompatibility group in this study and six complex IS*26*-class 1 integron arrangement patterns were distributed in 21 IncHI2-positive *Salmonella* isolates (Table 2). Thus, pDLST was performed to better characterize these IncHI2 plasmids. All IncHI2 plasmids were assigned to ST3 except one untypable IncHI2 plasmid in SJTUF 10456 due to a failure to detect the *smr0199* locus (data not shown), suggesting the occurrence of a new variant after multiple recombination events (Campos et al., 2016).

**Figure 4:**
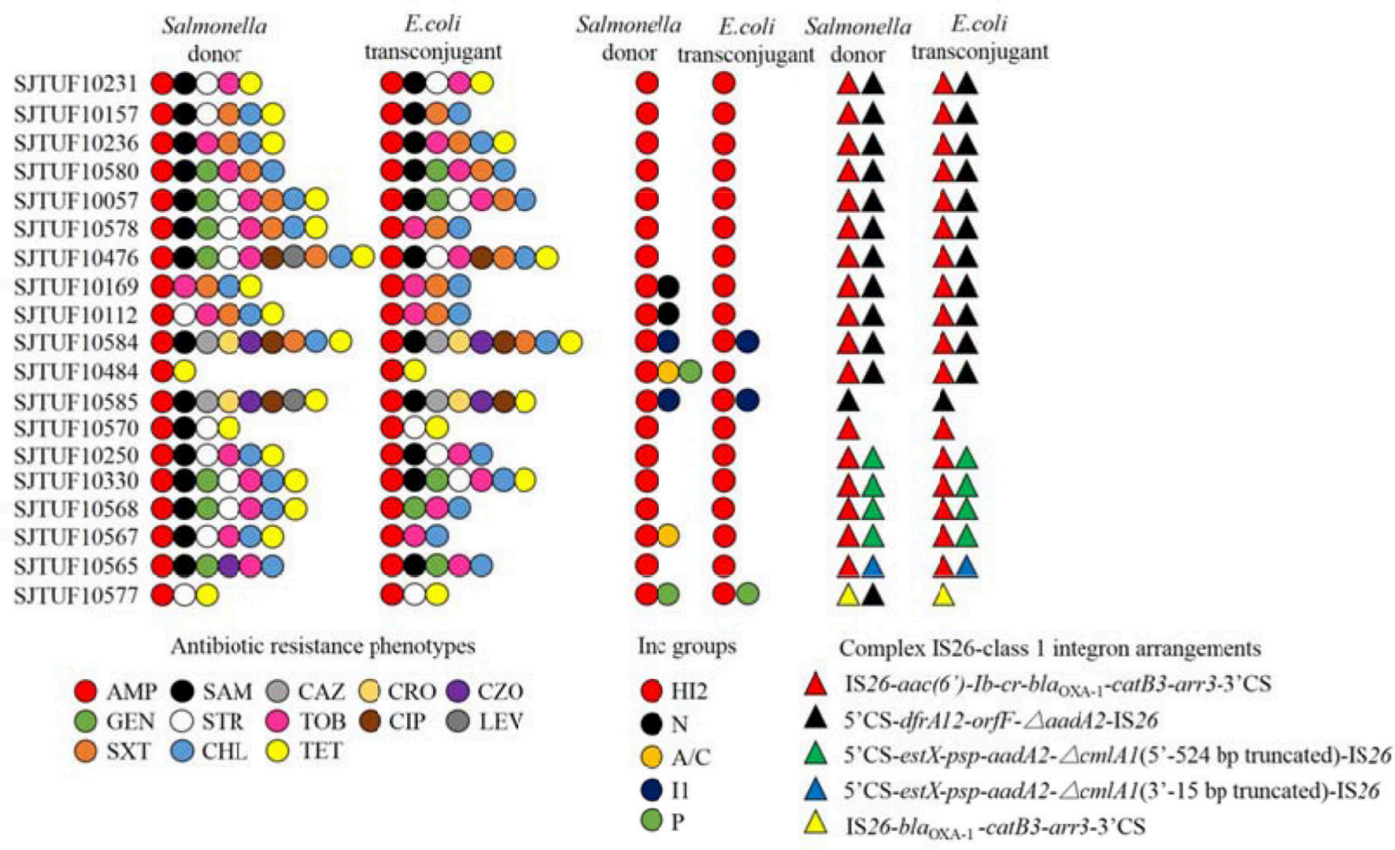
Comparison of *Salmonella* donors and the corresponding *E. coli* transconjugants based on antibiotic resistance profiles, plasmid replicon types and complex IS*26*-class 1 integron arrangement patterns. The antibiotics listed are abbreviated as follows: AMP, ampicillin; SAM, ampicillin/sulbactam; CAZ, ceftazidime; CRO, ceftriaxone; CZO, cephazolin; GEN, gentamicin; STR, streptomycin; TOB, tobramycin; CIP, ciprofloxacin; LEV, levofloxacin; SXT, sulfamethoxazole/trimethoprim; CHL, chloramphenicol; TET, tetracycline.

**Figure 5:**
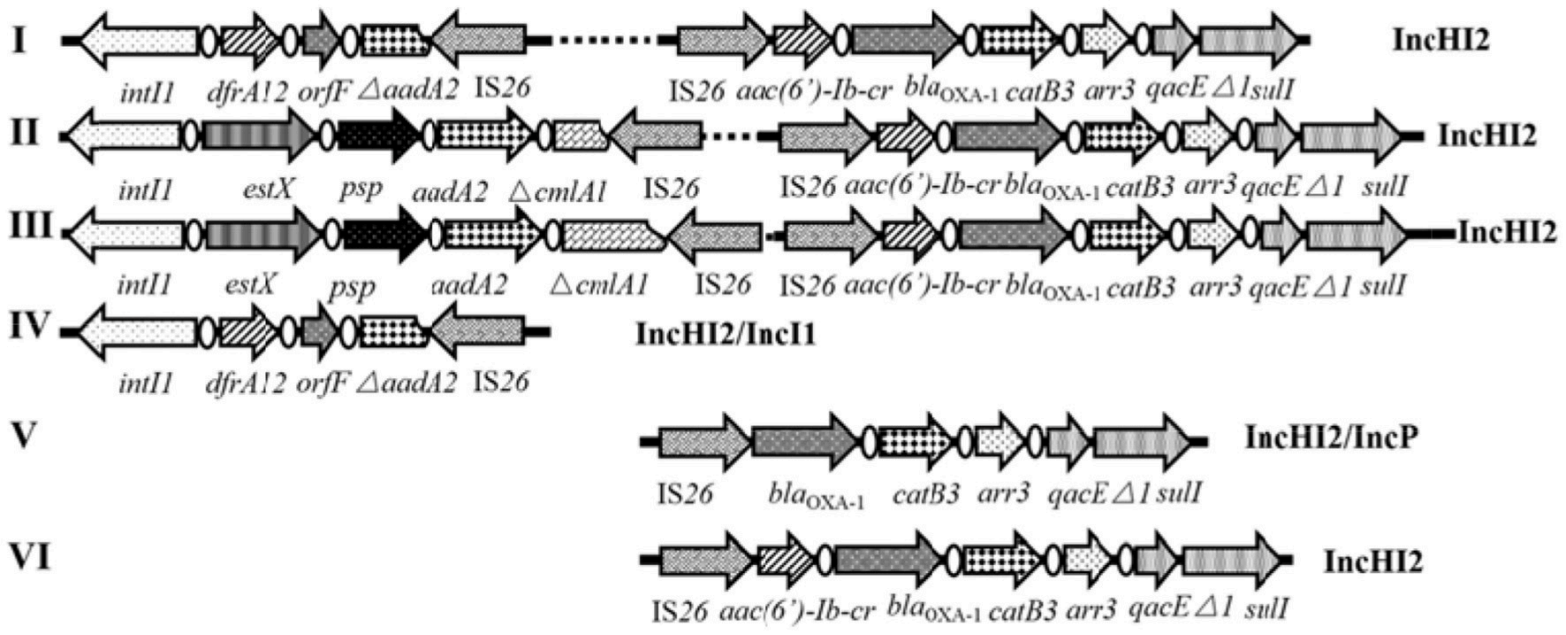
Schematic representation of IncHI2-associated complex IS*26*-class 1 integron arrangement patterns. The dotted lines imply omissions of the IncHI2 backbones. The orientation of each gene and insertion element is indicated by arrows.

Twenty-one isolates both harboring IncHI2 plasmid and complex IS*26*-class 1 integron arrangement were selected in the liquid mating assay to disclose their correlation. Nineteen transconjugants were obtained (Figure 4), and the conjugation rate was 90.5% (19/21). Based on the PCR-based replicon typing, IncHI2 plasmids from donors were all transferred to the *E. coli* rifampicin-resistant recipient with the co-transfer of IncI1 plasmids in SJTUF 10584 and SJTUF 10585 as well as IncP plasmid in SJTUF 10577. All of the detected complex IS*26*-class 1 integron arrangements were also transferred to the recipient after conjugation experiment except the genetic arrangement of 5′CS-*dfrA12-orfF-*Δ*aadA2*-IS*26* in SJTUF 10577. Six complex IS*26*-class 1 integron arrangement patterns associated with IncHI2 plasmids were further confirmed (Figures 4, 5) harboring 5 types of IS*26*-class 1 integron arrangements (corresponding to five probes I-V in Table 1). In addition, MDR phenotypes were also observed in the transconjugants, indicating that the majority of the MDR traits were determined by these ST3-IncHI2 plasmids. The conjugation frequencies of IncHI2 plasmids transferred alone were 10^−5^-10^−6^ (Table 3), reflecting that different complex IS*26*-class 1 integron arrangement patterns located on the IncHI2 plasmids didn't significantly affect conjugation frequencies (*P* > 0.05). IncHI2 and IncI1 plasmids were co-transferred with the conjugation frequencies of 10^−4^, reflecting that IncI1 plasmids could highly promote the co-transfer of IncHI2 plasmids.

**Table 3:** Conjugation frequencies of plasmids from eleven antibiotic-resistant *Salmonella* isolates and the resulting transferred plasmid incompatibility groups and complex IS*26*-class 1 integron arrangements.

**Isolate**	**IS*26*(→)-3′CS**	**5′CS-IS*26*(←)**	**Replicon**	**Conjugation frequency**
SJTUF 10236	IS*26-aac(6′)-Ib-cr*-*bla*_OXA−1_-*catB3*-*arr3-*3′CS	5′CS-*dfrA12*-*orfF*-*ΔaadA2*-IS*26*	HI2	6.100 × 10^−5^
SJTUF 10580	IS*26-aac(6′)-Ib-cr*-*bla*_OXA−1_-*catB3*-*arr3-*3′CS	5′CS-*dfrA12*-*orfF*-*ΔaadA2*-IS*26*	HI2	9.027 × 10^−6^
SJTUF 10476	IS*26-aac(6′)-Ib-cr*-*bla*_OXA−1_-*catB3*-*arr3-*3′CS	5′CS-*dfrA12*-*orfF*-*ΔaadA2*-IS*26*	HI2	5.682 × 10^−6^
SJTUF 10570	IS*26-aac(6′)-Ib-cr*-*bla*_OXA−1_-*catB3*-*arr3-*3′CS	ND	HI2	1.397 × 10^−6^
SJTUF 10330	IS*26-aac(6′)-Ib-cr*-*bla*_OXA−1_-*catB3*-*arr3-*3′CS	5′CS-*estx-psp-aadA2-ΔcmlA1(*5′-524 bp truncated)-IS*26*	HI2	1.704 × 10^−5^
SJTUF 10565	IS*26-aac(6′)-Ib-cr*-*bla*_OXA−1_-*catB3*-*arr3-*3′CS	5′CS*-estx-psp-aadA2-ΔcmlA1(*3′-15 bp truncated)-IS*26*	HI2	4.364 × 10^−5^
SJTUF 10577	*IS26-bla*_OXA−1_*-catB3-arr3-*3′CS	ND	HI2, P	9.412 × 10^−7^
SJTUF 10584	IS*26-aac(6′)-Ib-cr*-*bla*_OXA−1_-*catB3*-*arr3-*3′CS	5′CS-*dfrA12*-*orfF*-*ΔaadA2*-IS*26*	HI2, I1	6.774 × 10^−4^
SJTUF 10585	ND	5′CS-*dfrA12*-*orfF*-*ΔaadA2*-IS*26*	HI2, I1	3.226 × 10^−4^
SJTUF 10584	ND	ND	I1	6.404 × 10^−1^
SJTUF 10585	ND	ND	I1	2.230 × 10^−1^
SJTUF 10713	IS*26-ΔtnpR-tnpM-intI1-dfrA17-aadA5-*3′CS	5′CS-*dfrA12*-*orfF*-*ΔaadA2*-IS*26*	FIA, FIB	2.250 × 10^−6^
SJTUF 10718	ND	ND	FIIS	2.897 × 10^−7^

*Pink, IS26-blaOXA-1-catB3-arr3-3′CS; green, IS26-aac(6′)-Ib-cr-blaOXA-1-catB3-arr3-3′CS; red, 5′CS-dfrA12-orfF-ΔaadA2-IS26; blue, IS26-ΔtnpR-tnpM-intI1-dfrA17-aadA5-3'CS; purple, 5′CS-estX-psp-aadA2-ΔcmlA1(5′-524 bp truncated)-IS26; gray, 5′CS-estX-psp-aadA2-ΔcmlA1(3'-15 bp truncated)-IS26*.

Six tranconjugants (SJTUF10565-TC, SJTUF10568-TC, SJTUF10570-TC, SJTUF10577-TC SJTUF 10584-TC, and SJTUF10585-TC) covering the six typical IncHI2-associated complex IS*26*-class 1 integron arrangement patterns were selected for the analysis of S1-PFGE and Southern blot to confirm the genome-independent existence of IncHI2 plasmids and the localization of IS*26*-class 1 integron arrangements on IncHI2 plasmids. It is expected that SJTUF10565-TC harbors pattern III (probes II and V), SJTUF10568-TC harbors pattern II (probes II and IV), SJTUF10570-TC harbors pattern VI (probe II), SJTUF10577-TC harbors pattern V (probe I), SJTUF 10584-TC harbors pattern I (probes II and III), and SJTUF10585-TC harbors pattern IV (probe III) as shown in Figures 4, 5 and Table 1.

**Figure 6:**
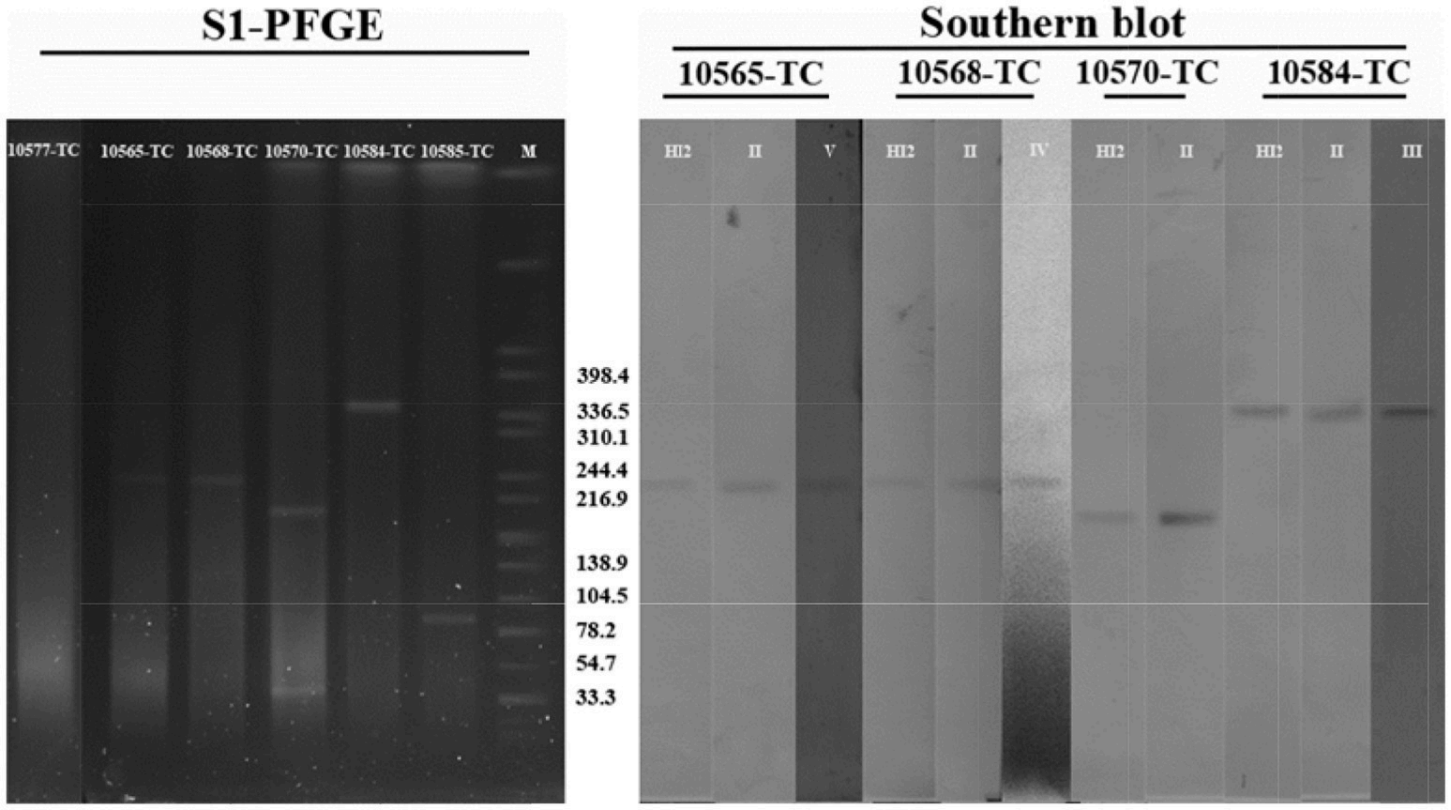
PFGE of S1-digested transconjugants DNA and Southern blot hybridization with specific IS*26*-class 1 integron arrangement probes shown in Table 1. M, *S*. Braenderup H9812 universal size standard.

S1-PFGE and subsequent Southern hybridization against DIG-labeled IncHI2 specific probes revealed that the size of IncHI2 plasmids in transconjugants with different IS*26*-class 1 integron arrangements ranged between 200 and 340 kb (Figure 6). It is noteworthy that there were no separated plasmids in SJTUF10577-TC which is inconsistent with previous PCR-based plasmid replicon typing results. This may be due to the nuclease degradation during S1 digestion or the plasmid integration into *E. coli* genome, which needs further study. There was only one plasmid in SJTUF 10584-TC and SJTUF10585-TC separated successfully by S1-PFGE, and the Southern blot confirmed only the plasmid in SJTUF 10584-TC belonged to IncHI2 group. The approximate 80-kb plasmid in SJTUF10585-TC possibly belonged to IncI1 group for that its size was in correspondence with the sequenced IncI1 plasmid (Tagg et al., 2014). Besides IncHI2 plasmid, there was one more untypable plasmid around 33 kb in SJTUF10570-TC. Therefore, except SJTUF 10577-TC and SJTUF 10585-TC, the four transconjugants SJTUF10565-TC, SJTUF10568-TC, SJTUF10570-TC, and SJTUF 10584-TC could be further involved in the Southern blot with different IS26-class 1 integron arrangement probes. As shown in Figure 6, the expected hybridization bands were displayed in all these four transconjugants with corresponding size of each IncHI2 plasmid. It could be concluded that the four IncHI2-associated complex IS*26*-class 1 integron arrangement patterns I-III, and VI (Figure 5) have been confirmed to locate on IncHI2 plasmids. Since SJTUF 10585-TC or SJTUF 10577-TC was the only transconjugant harboring IncHI2-associated complex IS*26*-class 1 integron arrangement pattern IV or V, it was hard to prove the localization of the pattern IV or V on IncHI2 plasmids in this study. Further whole genome sequencing including plasmids may be needed.

ST3-IncHI2 plasmids have been disseminated in multiple chicken and livestock farms of China, which are frequently associated with *fosA3* and *oqxAB* flanking by IS*26* (Yang et al., 2014; Fang et al., 2016; Wong et al., 2016). Despite of a recent study discovering the complex IS*26*-class 1 integron arrangement pattern I(i.e., IS*26*-*aac(6*′*)-Ib-cr*-*bla*_OXA−1_-*catB3-arr3*-3′CS plus 5′CS-*dfrA12-orfF-*Δ*aadA2*-IS*26*) through whole genome sequencing in two ST3-IncHI2 plasmids (Wong et al., 2016), few studies have systematically reported the association between ST3-IncHI2 plasmids and complex IS*26*-class 1 integron arrangements. The genetic arrangement of IS*26*-*aac(6*′*)-Ib-cr*-*bla*_OXA−1_-*catB3-arr3*-3′CS was the most popular IS*26*-class 1 integron arrangement identified in this study, with prevalent distribution in *S*. Typhimurium. The identical genetic context of the class 1 integron associated with IS*26* on IncHI2 plasmids was also detected in *S*. Indiana in China (Lai et al., 2013) as well as *S*. Typhimurium in Europe (Campos et al., 2016), suggesting a similar evolutionary origin and highlighting the potentially global spread of IncHI2 plasmids among *Salmonella*. Furthermore, a similar genetic context of that was also identified on an IncR plasmid of a *Klebsiella oxytoca* strain in Spain (Ruiz et al., 2011), and an IncN plasmid of an *E. coli* isolate in Hong Kong (Ho et al., 2013). The similar genetic module infers that the transfer of composite IS*26*-class 1 integron element between different plasmid replicons was probably mediated by IS*26* (Li L. et al., 2013; He et al., 2015). Moreover, the *sul3*-type class 1 integron with the array of *intI1*-*dfrA12-orfF-aadA2-cmlA1-aadA1-qacH-*IS*440-sul3* has been identified on IncA/C, IncI1 and IncB/O plasmids (Curiao et al., 2011; García et al., 2011), but rarely on IncHI2 plasmids. Sequence alignments revealed that two genetic arrangements of 5′CS*-estX-psp-aadA2-*Δ*cmlA1-*IS*26* identified on IncHI2 plasmids in this study may both originate from that *sul3*-type class 1 integron, indicating that IS*26* plays an important role in the transfer of *sul3*-type class 1 integrons between different plasmid replicons as well as the diversity of complex IS*26*-class 1 integron arrangements.

IncHI2 plasmids always show very conserved and stable scaffolds (García-Fernández and Carattoli, 2010), the formation of diverse complex IS*26*-class 1 integron arrangements may be mediated by IS*26* via a replicative mechanism or a TU element (Harmer et al., 2014; Harmer and Hall, 2015; He et al., 2015). The transposition activity of IS*26* collaborates with capture and integration of class 1 integrons, resembling resistance gene clusters onto a single plasmid. IS*26*-mediated further fusion and reorganization of such plasmids will facilitate the occurrence of novel IncHI2 derivative plasmids with various MDR regions (He et al., 2015; Fang et al., 2016; García et al., 2016). In addition, gene cassettes embedded in atypical class 1 integrons with 5′CS or 3′CS interrupted by IS*26* couldn't be screened by conventional PCR. Thus, the comprehensive study of complex IS*26*-class 1 integron arrangements in *Salmonella* may provide a new perspective in tracing the spread and evolution of IS*26*-class 1 integron-mediated MDR as well as MDR IncHI2 plasmids.

The conjugation frequencies of IncHI2 plasmids transferred alone at 37°C ranged from 10^−5^ to 10^−6^ (Table 3), and different complex IS*26*-class 1 integron arrangement patterns located on the IncHI2 plasmids didn't significantly affect conjugation frequencies (*P* > 0.05), inferring that conjugation frequency may mainly depend on the mutual regulation by transfer-related functional elements located on the plasmid (Page et al., 1999; Gruber et al., 2016). The conjugation frequency of IncI1 plasmids transferred alone was 10^−1^, and then became 10^−4^ while co-transfer with IncHI2 plasmids, reflecting that highly conjugative plasmids could promote the movement of other plasmids. García et al. (2007) found that the conjugation frequency of IncHI2 plasmids transferred together with IncF plasmids increased two orders of magnitude than that of IncHI2 plasmids transferred alone, attributing to the formation of cointegrate FIB–HI2 plasmid fusion during transfer process. Whether IncI1 plasmids promoted the transfer of IncHI2 plasmids through a similar mechanism may need further study. IncHI2 plasmids belonging to broad-host-range plasmid vectors contain several functional elements to ensure their stable permanence in host, such as the mutagenesis induction system (*mucAB*), the *relE/relB* toxin–antitoxin system and bacteriophage inhibition (*phi*) (García-Fernández and Carattoli, 2010). Meanwhile, IncHI2 plasmids exhibit optimal transfer capacity at low temperature (<30°C), along with co-transfer driven by highly conjugative plasmids like IncI1 plasmids, will facilitate the dissemination of composite IS*26*-class 1 integron MDR elements among various bacterial species. Thus, increased active surveillance of the MDR IncHI2 plasmids carrying such composite IS*26*-class 1 integron elements in *Salmonella* is urgently needed.

In conclusion, class 1 integrons represent the hot spot for IS*26* insertion, and IS*26* can insert into class 1 integrons at different sites, forming diverse MDR loci. Moreover, ST3-IncHI2 was the major plasmid lineage contributing to the horizontal transfer of composite IS*26*-class 1 integron MDR elements. Further investigation of complex IS*26*-class 1 integron arrangements is urgently needed to track and monitor the spread of IS*26*-class 1 integron-mediated antibiotic resistance as well as the evolution of IncHI2 MDR plasmids.

## Author contributions

HZ completed the screening of IS26-positive isolates, detection of Class 1 integrons, genetic context analysis of Class 1 integrons associated with IS26, conjugation experiments, and IncHI2 plasmid characterization. WC completed the isolate collection, antimicrobial susceptibility test, screening of IS26-positiveisolates, and detection of Class 1 integrons. XX helped to finish the isolate collection. XZ helped to finish the isolate collection, antimicrobial susceptibility test, and data release. CS designed the project, completed the data analysis, and prepared the manuscript.

### Conflict of interest statement

The authors declare that the research was conducted in the absence of any commercial or financial relationships that could be construed as a potential conflict of interest.
