# Supplementary material for: Transmissible ST3-IncHI2 Plasmids Are Predominant Carriers of Diverse Complex IS26-Class 1 Integron Arrangements in Multidrug-Resistant Salmonella
Source: Front Microbiol. 2018 Oct 23;9:2492. doi: 10.3389/fmicb.2018.02492 (PMC6206278; doi:10.3389/fmicb.2018.02492)
Supplement: Supplementary file 1 [file Table_1.DOCX]

Supplementary Material

Transmissible ST3-IncHI2 Plasmids Are Predominant Carriers of Diverse Complex IS*26*-Class 1 Integron Arrangements in Multidrug-Resistant *Salmonella*

Hang Zhao†, Wenyao Chen†, Xiujuan Zhou, Chunlei Shi*

MOST-USDA Joint Research Center for Food Safety, School of Agriculture and Biology, and State Key Laboratory of Microbial Metabolism, Shanghai Jiao Tong University, Shanghai 200240, China

**†**These authors have contributed equally to the work.

*** Correspondence:**Chunlei Shi, MOST-USDA Joint Research Center for Food Safety, School of Agriculture and Biology, and State Key Laboratory of Microbial Metabolism, Shanghai Jiao Tong University, Shanghai 200240, China
[clshi@sjtu.edu.cn](mailto:clshi@sjtu.edu.cn)

**Table S1.** *Salmonella* isolates used in this study (n=74).

| **Strain** | **Serovar** | | **Year** | | **Region** | | **Sample type** | | **Plasmid replicon** | |
| --- | --- | --- | --- | --- | --- | --- | --- | --- | --- | --- |
| SJTUF10057 | Typhimurium | | 2006 | | Shanghai | | Feces | | HI2 | |
| SJTUF10112 | Typhimurium | | 2006 | | Shanghai | | Feces | | HI2、N | |
| SJTUF10155 | Typhimurium | | 2006 | | Shanghai | | Feces | | HI2、P、N、FIIs、FIC | |
| SJTUF10157 | Typhimurium | | 2006 | | Shanghai | | Feces | | HI2 | |
| SJTUF10169 | Typhimurium | | 2006 | | Shanghai | | Feces | | HI2、N | |
| SJTUF10211 | Typhimurium | | 2007 | | Shanghai | | Feces | | HI2、P | |
| SJTUF10231 | Typhimurium | | 2007 | | Shanghai | | Feces | | HI2 | |
| SJTUF10233 | Typhimurium | | 2007 | | Shanghai | | Feces | | HI2、P、FIIs | |
| SJTUF10236 | Typhimurium | 2007 | | Shanghai | | Feces | | HI2 | |  |
| SJTUF10250 | Typhimurium | | 2007 | | Shanghai | | Feces | | HI2 | |
| SJTUF10327 | Typhimurium | | 2007 | | Shanghai | | Feces | | A/C | |
| SJTUF10328 | Typhimurium | | 2007 | | Shanghai | | Feces | | HI2、P、N、FIIs | |
| SJTUF10329 | Typhimurium | | 2007 | | Shanghai | | Feces | | HI2、P、N、FIIs | |
| SJTUF10330 | Typhimurium | | 2007 | | Shanghai | | Feces | | HI2 | |
| SJTUF10479 | Typhimurium | | 2007 | | Shanghai | | Chicken | | HI2 | |
| SJTUF10484 | Typhimurium | | 2007 | | Shanghai | | Clam | | HI2、P、A/C | |
| SJTUF10565 | Typhimurium | | 2006 | | Shanghai | | Chicken | | HI2 | |
| SJTUF10567 | Typhimurium | | 2006 | | Shanghai | | Pork | | HI2、A/C | |
| SJTUF10568 | Typhimurium | | 2006 | | Shanghai | | Pork | | HI2 | |
| SJTUF10570 | Typhimurium | | 2006 | | Shanghai | | Pork | | HI2 | |
| SJTUF10577 | Typhimurium | | 2006 | | Shanghai | | Saury | | HI2 | |
| SJTUF10578 | Typhimurium | | 2006 | | Shanghai | | Pork | | HI2 | |
| SJTUF10586 | Typhimurium | | 2006 | | Shanghai | | Chicken | | HI2、I1 | |
| SJTUF10694 | Typhimurium | | 2006 | | Wuhan | | Beef | | HI2、P、FIIs | |
| SJTUF10024 | Enteritidis | | 2006 | | Shanghai | | Feces | | P、N、FIIs | |
| SJTUF10029 | Enteritidis | | 2006 | | Shanghai | | Feces | | P、N、FIIs | |
| SJTUF10229 | Enteritidis | | 2007 | | Shanghai | | Feces | | FIIs | |
| SJTUF10331 | Enteritidis | | 2007 | | Shanghai | | Feces | | P、FIIs | |
| SJTUF10459 | Enteritidis | | 2007 | | Shanghai | | Carrot | | P、N、FIIs | |
| SJTUF10462 | Enteritidis | | 2007 | | Shanghai | | Chicken | | P、N | |
| SJTUF10491 | Enteritidis | | 2007 | | Shanghai | | Chicken | | P、N | |
| SJTUF10571 | Enteritidis | | 2006 | | Shanghai | | Chicken | | P、FIIs | |
| SJTUF10587 | Enteritidis | | 2006 | | Shanghai | | Duck | | P、FIIs | |
| SJTUF10717 | Enteritidis | | 2006 | | Wuhan | | Feces | | P、FIIs、I1 | |
| SJTUF10718 | Enteritidis | | 2006 | | Wuhan | | Feces | | FIIs | |
| SJTUF10720 | Enteritidis | | 2006 | | Wuhan | | Feces | | P、FIIs | |
| SJTUF10119 | Braenderup | | 2006 | | Shanghai | | Feces | | HI2、P、N | |
| SJTUF10334 | Braenderup | | 2007 | | Shanghai | | Feces | | P、N、FIC | |
| SJTUF10207 | Indiana | | 2007 | | Shanghai | | Feces | | P、N | |
| **Strain** | **Serovar** | | **Year** | | **Region** | | **Sample type** | | **Plasmid replicon** | |
| SJTUF10476 | Indiana | | 2007 | | Shanghai | | Chicken | | HI2 | |
| SJTUF10566 | Indiana | | 2006 | | Shanghai | | Chicken | | P | |
| SJTUF10584 | Indiana | | 2006 | | Shanghai | | Chicken | | HI2、I1 | |
| SJTUF10585 | Indiana | | 2006 | | Shanghai | | Chicken | | HI2、I1 | |
| SJTUF10702 | Indiana | | 2006 | | Wuhan | | Chicken | | P | |
| SJTUF10713 | Heidelberg | | 2006 | | Wuhan | | Chicken | | FIA、FIB | |
| SJTUF10740 | Heidelberg | | 2006 | | Wuhan | | Chicken | | P | |
| SJTUF10772 | Heidelberg | | 2006 | | Wuhan | | Feces | | P | |
| SJTUF10456 | Derby | | 2007 | | Shanghai | | Pork | | HI2、P、N、FIC | |
| SJTUF10469 | Derby | | 2007 | | Shanghai | | Pork | | P、FIC | |
| SJTUF10475 | Derby | | 2007 | | Shanghai | | Pork | | P、FIC、I1 | |
| SJTUF10560 | Derby | | 2006 | | Shanghai | | Pork | | P | |
| SJTUF10589 | Derby | | 2006 | | Shanghai | | Pork | | P、FIC | |
| SJTUF10754 | Derby | | 2006 | | Wuhan | | Feces | | HI2、P | |
| SJTUF10482 | Anatum | | 2007 | | Shanghai | | Pork | | I1 | |
| SJTUF10580 | Anatum | | 2006 | | Shanghai | | Pork | | HI2 | |
| SJTUF10762 | Anatum | | 2006 | | Wuhan | | Feces | | P | |
| SJTUF10230 | Agona | | 2007 | | Shanghai | | Feces | | HI2、P、N | |
| SJTUF10247 | Agona | | 2007 | | Shanghai | | Feces | | P、N | |
| SJTUF10249 | Agona | | 2007 | | Shanghai | | Feces | | P、N、FIIs | |
| SJTUF10725 | Agona | | 2007 | | Wuhan | | Feces | | P、N | |
| SJTUF10750 | Manhattan | | 2007 | | Wuhan | | Pork | | N | |
| SJTUF10213 | Thompson | | 2007 | | Shanghai | | Feces | | P | |
| SJTUF10703 | Thompson | | 2007 | | Shanghai | | Shrimp | | A/C | |
| SJTUF10051 | Aberdeen | | 2006 | | Shanghai | | Feces | | HI2、P | |
| SJTUF10701 | Infantis | | 2007 | | Wuhan | | Pork | | P | |
| SJTUF10782 | Kentucky | | 2007 | | Wuhan | | Pork | | P、N | |
| SJTUF10768 | Litchfield | | 2007 | | Wuhan | | Feces | | P | |
| SJTUF10483 | Mbandaka | | 2007 | | Shanghai | | Pork | | P | |
| SJTUF10216 | Montevideo | | 2007 | | Shanghai | | Feces | | A/C | |
| SJTUF10721 | Paratyphi A | | 2007 | | Wuhan | | Feces | | P | |
| SJTUF10485 | Paratyphi B | | 2007 | | Shanghai | | Pork | | P | |
| SJTUF10573 | Stanley | | 2006 | | Shanghai | | Razor clam | | P | |
| SJTUF10700 | Virchow | | 2007 | | Wuhan | | Orange juice | | P | |
| SJTUF10705 | Typhi | | 2007 | | Wuhan | | Feces | | P | |

**Table S2.** Oligonucleotides primers for the detection of class 1 integrons and their genetic contexts associated with IS*26*

| **Target** | **Primer** | **Nucleotide sequence (5’→3’)** | **Reference** |
| --- | --- | --- | --- |
| *intI1* | *intI1*-F  *intI1*-R | GCTTCGTGATGCCTGCTTG  GCTGCGTTCGGTCAAGGT | This study  This study |
| *qacE△1-sulI* | QS-F  QS-R | TTCTGAAATCCATCCCTGTCGGTGTTGC  CAAGGCTCGCTGGACCCAGATCCTTTA | This study  This study |
| Variable region | 5’CS  *qacE△1*R | GGCATCCAAGCAGCAAGC  CAAGCTTTTGCCCATGAAGC | [Casella et al. (2015)](#_ENREF_2)  [Casella et al. (2015)](#_ENREF_2) |
| Variable region | 5’CS  3’CS | GGCATCCAAGCAGCAAGC  AAGCAGACTTGACCTGAT | [Casella et al. (2015)](#_ENREF_2)  [Casella et al. (2015)](#_ENREF_2) |
| Variable region | hep58  hep59 | TCATGGCTTGTTATGACTGT  GTAGGGCTTATTATGCACGC | [Malek et al. (2015)](#_ENREF_3)  [Malek et al. (2015)](#_ENREF_3) |
| Variable region | 5’CS  hep59 | GGCATCCAAGCAGCAAGC  GTAGGGCTTATTATGCACGC | [Casella et al. (2015)](#_ENREF_2)  [Malek et al. (2015)](#_ENREF_3) |
| IS*26*-integron | HS1081  *qacE△1R* | AATGCGCCTGGTAAGCAGAG  CAAGCTTTTGCCCATGAAGC | [Betteridge et al. (2011)](#_ENREF_1)  [Casella et al. (2015)](#_ENREF_2) |
| Integron-IS*26* | 5’CS  IS*26*-F | GGCATCCAAGCAGCAAGC  ATGAACCCATTCAAAGGCCG | [Casella et al. (2015)](#_ENREF_2)  [Rodríguez-Martínez et al. (2013)](#_ENREF_4) |
| Integron-IS*26* | 5’CS  IS*26*-3-F | GGCATCCAAGCAGCAAGC  GAATGCGATCATGGCAAACTGAAACG | [Casella et al. (2015)](#_ENREF_2)  This study |

Betteridge, T., Partridge, S.R., Iredell, J.R., and Stokes, H.W. (2011). Genetic Context and Structural Diversity of Class 1 Integrons from Human Commensal Bacteria in a Hospital Intensive Care Unit. *Antimicrob. Agents Chemother.* 55(8)**,** 3939-3943. doi: 10.1128/AAC.01831-10.

Casella, T., Rodríguez, M.M., Takahashi, J.T., Ghiglione, B., Dropa, M., Assunção, E., et al. (2015). Detection of *bla*_CTX-M_-type genes in complex class 1 integrons carried by *Enterobacteriaceae* isolated from retail chicken meat in Brazil. *Int. J. Food Microbio.* 197**,** 88-91. doi: <http://dx.doi.org/10.1016/j.ijfoodmicro.2014.12.001>.

Malek, M.M., Amer, F.A., Allam, A.A., El-Sokkary, R.H., Gheith, T., and Arafa, M.A. (2015). Occurrence of classes I and II integrons in *Enterobacteriaceae* collected from Zagazig University Hospitals, Egypt. *Front. Microbiol.* 6**,** 601. doi: 10.3389/fmicb.2015.00601.

Rodríguez-Martínez, J.M., Díaz de Alba, P., Briales, A., Machuca, J., Lossa, M., Fernández-Cuenca, F., et al. (2013). Contribution of OqxAB efflux pumps to quinolone resistance in extended-spectrum-*β*-lactamase-producing *Klebsiella pneumoniae*. *J. Antimicrob. Chemother.* 68(1)**,** 68-73. doi: 10.1093/jac/dks377.
